# IL-33 and Superantigenic Activation of Human Lung Mast Cells Induce the Release of Angiogenic and Lymphangiogenic Factors

**DOI:** 10.3390/cells10010145

**Published:** 2021-01-12

**Authors:** Leonardo Cristinziano, Remo Poto, Gjada Criscuolo, Anne Lise Ferrara, Maria Rosaria Galdiero, Luca Modestino, Stefania Loffredo, Amato de Paulis, Gianni Marone, Giuseppe Spadaro, Gilda Varricchi

**Affiliations:** 1 Department of Translational Medical Sciences, University of Naples Federico II, 80131 Naples, Italy; l.cristinziano@gmail.com (L.C.); remo.poto@gmail.com (R.P.); gjada.criscuolo2@unina.it (G.C.); Anneliseferrara@gmail.com (A.L.F.); mrgaldiero@libero.it (M.R.G.); modestinoluca@gmail.com (L.M.); stefanialoffredo@hotmail.com (S.L.); depaulis@unina.it (A.d.P.); marone@unina.it (G.M.); spadaro@unina.it (G.S.); 2 World Allergy Organization (WAO) Center of Excellence, 80131 Naples, Italy; 3 Center for Basic and Clinical Immunology Research (CISI), University of Naples Federico II, 80131 Naples, Italy; 4 Institute of Experimental Endocrinology and Oncology (IEOS), National Research Council, 80131 Naples, Italy

**Keywords:** allergy, asthma, histamine, IL-33, mast cell, protein A, protein L, superantigen, VEGF-A, VEGF-C

## Abstract

Human lung mast cells (HLMCs) express the high-affinity receptor FcεRI for IgE and are strategically located in different compartments of human lung, where they play a role in several inflammatory disorders and cancer. Immunoglobulin superantigens (e.g., protein A of *Staphylococcus aureus* and protein L of *Peptostreptococcus magnus*) bind to the variable regions of either the heavy (V_H_3) or light chain (κ) of IgE. IL-33 is a cytokine expressed by epithelial cells that exerts pleiotropic functions in the lung. The present study investigated whether immunoglobulin superantigens protein A and protein L and IL-33 caused the release of inflammatory (histamine), angiogenic (VEGF-A) and lymphangiogenic (VEGF-C) factors from HLMCs. The results show that protein A and protein L induced the rapid (30 min) release of preformed histamine from HLMCs. By contrast, IL-33 did not induce the release of histamine from lung mast cells. Prolonged incubation (12 h) of HLMCs with superantigens and IL-33 induced the release of VEGF-A and VEGF-C. Preincubation with IL-33 potentiated the superantigenic release of histamine, angiogenic and lymphangiogenic factors from HLMCs. Our results suggest that IL-33 might enhance the inflammatory, angiogenic and lymphangiogenic activities of lung mast cells in pulmonary disorders.

## 1. Introduction

Mast cells, localized in different compartments of human lung [1,2,3,4], are critical sentinels in immunity [5,6]. Mast cells were canonically considered primary effector cells of allergic disorders [2,7,8,9]. There is now evidence that these cells play a role in bacterial and viral infections [6,10,11,12], pulmonary disorders [13], angiogenesis [14,15,16,17], lymphangiogenesis [18,19], autoimmune diseases [20,21,22], and cancer [23,24,25,26].

Human lung mast cells express the high-affinity receptor (FcεRI) for immunoglobulin E [1,27,28]. IgE is a heterotetramer consisting of two identical heavy chains and two identical light chains that bind with high affinity (Ka ≅ 10^10^ M^−1^) to FcεRI on mast cells [29]. The human FcεRI is a tetrameric complex comprising a single α chain, responsible for binding to IgE, two disulfide-linked γ chains and a single β chain [30,31]. Aggregation of IgE/FcεRI complex by multivalent antigen, divalent anti-IgE or anti-FcεRI initiates human mast cell activation [32,33] and the release of preformed (e.g., histamine), de novo synthesized lipid mediators [e.g., prostaglandin D_2_ (PGD_2_) and cysteinyl leukotriene C_4_ (LTC_4_)], chemokines [34,35] and cytokines [8]. Human lung mast cells [14], like macrophages [36], basophils [37], and neutrophils [38], also release angiogenic (e.g., vascular endothelial growth factor A: VEGF-A) and lymphangiogenic factors (e.g., vascular endothelial growth factor C: VEGF-C) [7,14,36].

*Staphylococcus aureus* (*S. aureus*) is a multifaceted human pathobiont which synthesizes several T [39] and B cell superantigens (SAgs) (e.g., protein A) [40]. Most clinical isolates of *S. aureus* synthesize and release protein A [41] which has two binding sites for human immunoglobulins (Igs): the classical site binds Fcγ [42], whereas the alternative site binds the Fab portion of 15% to 50% of human polyclonal IgG, IgM, IgA, and IgE [43]. In particular, the alternative site of protein A binds specifically to V_H_3, the largest of human Ig germline V_H_ domain of human Igs [44]. Protein L, synthesized by *Peptostreptococcus magnus* (*P. magnus*) is another SAg that binds to the V domain of the κ light chains of human Igs, including IgE [45,46,47,48]. In particular, protein L binds with high-affinity only human VkI, VkIII and VkIV subtypes, but does not interact with VkII subtype [49]. Several allergic [50,51,52,53,54,55] and autoimmune disorders [56,57], neoplasia [58,59,60], and immunodeficiencies [44] can be associated with SAgs.

Interleukin-33 (IL-33) is an IL-1 family member [61] expressed by lung epithelial and endothelial cells, and by other stromal cells [62,63,64]. IL-33, released after cellular stress or damage, acts as an alarmin that activates the immune response [65,66]. IL-33 binds to a heterodimer formed by its primary receptor ST2 and the co-receptor IL-1 receptor accessory protein (IL1RAP). Engagement of IL-33 receptor results in the release of mediators by different immune cells [67], including mast cells [17,22,68,69,70,71,72,73,74]. IL-33 is involved in allergic disorders [75,76,77,78], bacterial and viral infections [79,80,81,82] and cancer [66,83,84].

The aim of this study was to evaluate whether protein A and protein L, alone or in combination with IL-33, induce the release of inflammatory, angiogenic and lymphangiogenic factors from primary human lung mast cells.

## 2. Materials and Methods

### 2.1. Reagents

Bovine serum albumin (BSA), PIPES [piperazine-N,N′-bis (2-ethanesulfonic acid)], L-glutamine, antibiotic-antimycotic solution (10,000 IU penicillin, 10 mg/mL streptomycin, and 25 μg/mL amphotericin B), IL-33 (R & D Systems, Minneapolis, MN, USA), collagenase (Worthington Biochemical Co., Freehold, NJ, USA), fetal calf serum (FCS) (GIBCO, Grand Island, NY, USA), pronase (Calbiochem, La Jolla, CA, USA), RPMI 1640 with 25 mM HEPES buffer, Eagle’s minimum essential medium (Flow Laboratories, Irvine, UK), Percoll (Pharmacia Fine Chemicals, Uppsala, Sweden), and CD117 MicroBead (Miltenyi Biotech, Bologna, Italy) were obtained commercially. The monoclonal antibody (mAb) anti-FcεRI was a gift from Dr. Lawrence M. Lichtenstein (The Johns Hopkins University, Baltimore, MD, USA). Human IgG anti-IgE (H-aIgE) was purified from the serum of a patient with atopic dermatitis as previously described [85,86]. The specificity and activity of IgG anti-IgE were described elsewhere [85].

### 2.2. Human Monoclonal IgM and IgE and Human Polyclonal IgG

Monoclonal IgM, purified from the serum of patients with Waldenström’s macroglobulinemia, were described previously [87]. Variable regions of these monoclonal IgM were determined using a panel of primary sequence-dependent V_H_ family specific reagents that identify framework regions [88]. Human polyclonal IgG were purified from the serum of healthy donors [89]. Monoclonal IgE λ and κ, purified from the serum of patients with IgE myeloma, were described elsewhere [89,90].

### 2.3. Isolation of HLMCs

The study was approved by the Ethics Committee of the University of Naples Federico II (Protocol: Human MC No. 7/19). The lung tissue was obtained from patients who were seronegative for HIV-1, HCV, and HBV undergoing thoracic surgery. HLMCs were isolated from human lung tissue by a modification of the method previously described [14]. The enzymatic dispersion tissue yields ≈5 × 10^5^ mast cells per gram of lung tissue. The purity of these populations ranged from 3% to 18%. HLMCs were partially purified by flotation through a discontinuous Percoll gradient [87]. Mast cell purity using this technique ranged from 49% to 81% and was assessed by alcian blue staining.

### 2.4. Histamine Release

HLMCs (≈3 × 10^4^ mast cells per tube) were resuspended in PIPES buffer containing, in addition to PIPES (25 mM), CaCl_2_ (2 mM) and dextrose (1 g/L). 0.3 mL of the cell suspensions were placed in 12 × 75 mm polyethylene tubes. 0.2 mL of each prewarmed releasing stimulus was added, and incubation was continued at 37 °C for 45 min [91]. At the end of incubation, cells were centrifuged (1000× *g*, 4 °C, 5 min) and supernatants were stored at –20 °C for subsequent assay of histamine. Histamine was measured in duplicate determinations with an automated fluorometric technique [92].

### 2.5. VEGF-A and VEGF-C Release

HLMCs (≈8 × 10^4^ mast cells/per tube) were incubated (37 °C, 12 h) in RPMI 1640 containing 5% FCS, 2 mM L-glutamine, and 1% antibiotic-antimycotic solution, and activated with various concentrations of protein A or protein L, alone or in combination with IL-33. At the end of incubation, cells were centrifuged (1000× *g*, 4 °C, 5 min) and the supernatants were stored at −80 °C for subsequent assay of mediator release. VEGF-A and VEGF-C were measured in duplicate determinations using ELISA kits (R&D System, Minneapolis, MN, USA [93]. The ELISA sensitivity is 31–2000 pg/mL for VEGF-A and 62–4000 pg/mL for VEGF-C.

### 2.6. Statistical Analysis

Data were analyzed with the GraphPad Prim 6 software package (GraphPad Software, La Jolla, CA, USA). Values were expressed as mean ± SEM (standard error of the mean). Statistical analysis was performed using Student’s *t*-test or one-way analysis of variance [71]. Correlations between two variables were assessed by Spearman’s rank correlation analysis and reported as coefficients of correlation (r). A *p* value ≤ 0.05 was considered statistically significant.

## 3. Results

### 3.1. Effects of Human IgG Anti-IgE on the Release of Angiogenic and Lymphangiogenic Factors from HLMCs

**Figure 1:**
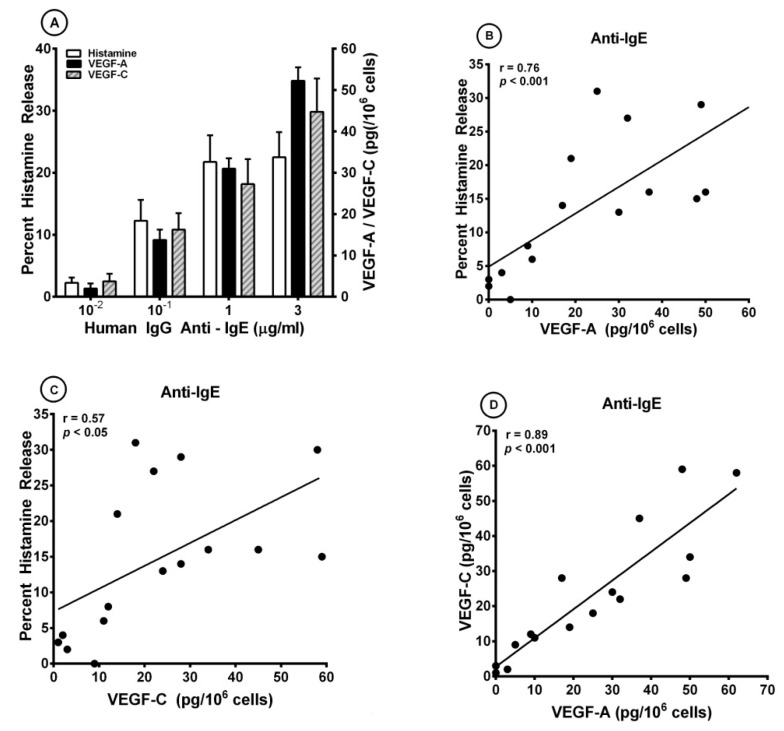
(**A**) Effects of increasing concentrations of human IgG anti-IgE purified from the serum of an atopic dermatitis patient [85,95] on histamine release and the production of VEGF-A and VEGF-C from four different preparations of human lung mast cells (HLMCs). HLMCs were incubated (45 min at 37 °C) with the indicated concentrations of IgG anti-IgE for histamine secretion or (12 h at 37 °C) for VEGF-A and VEGF-C release. Each bar is the mean ± SEM; (**B**) Correlation (r = 0.76; *p* < 0.001) between VEGF-A release and the percent histamine secretion caused by human IgG anti-IgE from HLMCs; (**C**) Correlation (r = 0.57; *p* < 0.05) between VEGF-C release and the percent histamine secretion caused by human IgG anti-IgE from HLMCs; (**D**) Correlation (r = 0.89; *p* < 0.001) between VEGF-A and VEGF-C release caused by human IgG anti-IgE from HLMCs.

We have previously reported that IgG anti-IgE purified from the serum of a small percentage of atopic dermatitis patients induced histamine release from human basophils [85] and lung mast cells [94]. The activating effects of human IgG anti-IgE (H-aIgE) were mediated by the interaction with membrane-bound IgE on human basophils and mast cells. In a series of experiments, we used this human autoantibody to activate HLMCs in vitro. H-aIgE (10^−2^ to 3 μg/mL) caused a concentration-dependent release of both angiogenic (VEGF-A) and lymphangiogenic factors (VEGF-C) from four different preparations for HLMCs (Figure 1A). As a control, we found that the same concentrations of H-aIgE induced a concentration-dependent release of histamine. Similar results were obtained when HLMCs were activated by increasing concentrations (10^−1^ to 3 μg/mL) of monoclonal antibody (mAb) anti-FcεRI (Table 1). Three preparations of human polyclonal IgG (10^−2^ to 3 μg/mL) did not cause the release of histamine, VEGF-A, and VEGF-C (Table 2). These results indicate that mast cells isolated from human lung parenchyma express IgE bound to FcεRI. Figure 1B shows that there was a significant correlation between the production of VEGF-A and histamine release caused by H-aIgE (r = 0.76; *p* < 0.001). Similarly, there was a significant correlation between the production of VEGF-C and histamine release (r = 0.57; *p* < 0.05) (Figure 1C) and between the production of angiogenic (VEGF-A) and lymphangiogenic (VEGF-C) factors (r = 0.89; *p* < 0.001) (Figure 1D).

### 3.2. Effect of Superantigenic Protein A on the Release of Angiogenic and Lymphangiogenic Factors from HLMCs

**Figure 2:**
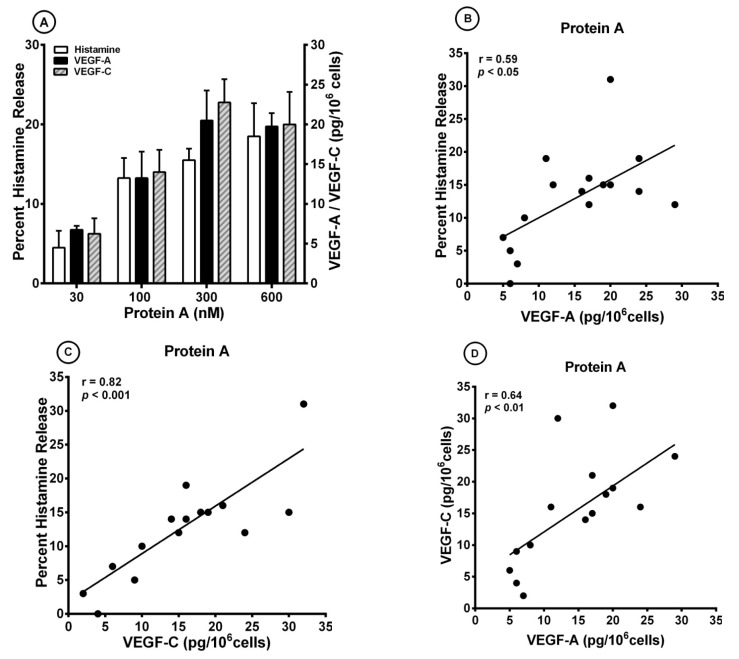
(**A**) Effects of increasing concentrations of protein A on histamine release and the production of VEGF-A and VEGF-C from four different preparations of human lung mast cells (HLMCs). HLMCs were incubated (45 min at 37 °C) with the indicated concentrations of protein A for histamine secretion or (12 h at 37 °C) for VEGF-A and VEGF-C release. Each bar is the mean ± SEM; (**B**) Correlation (r = 0.59; *p* < 0.05) between VEGF-A release and the percent histamine secretion caused by protein A from HLMCs; (**C**) Correlation (r = 0.82; *p* < 0.001) between VEGF-C release and the percent histamine secretion caused by protein A from HLMCs; (**D**) Correlation (r = 0.64; *p* < 0.01) between VEGF-A and VEGF-C release caused by protein A from HLMCs from HLMCs.

*S. aureus* colonization is associated with bronchial asthma [52,95]. *S. aureus* superantigens trigger airway inflammation and increased airway responsiveness, and facilitate allergic sensitization in asthma models [96]. It has been shown that *S. aureus* and protein A can activate human mast cells through different mechanisms [47,97]. More recently, we have demonstrated that protein A induced the release of lipid mediators from human cardiac mast cells through the engagement of IgE V_H_3^+^ bound to FcεRI [98]. Figure 2A shows that protein A (30 to 600 nM) caused a concentration-dependent release of both VEGF-A and VEGF-C from different preparations of HLMCs. The same concentrations of protein A caused a dose-dependent release of histamine. Protein A contains five homologous repeated domains, each of which binds to human Igs, including IgE [42,43]. Preincubation (15 min, 37 °C) of protein A (300 nM) with IgM V_H_3^+^ (10 μg/mL), but not IgM V_H_6^+^ (10 μg/mL), blocked the histamine-releasing activity of protein A (Table 3). These results suggest that the immunoglobulin superantigen protein A activates HLMCs through the binding to IgE V_H_3^+^ bound to FcεRI.

There was a significant correlation between the release of VEGF-A and histamine secretion (r = 0.59; *p* < 0.05) caused by protein A (Figure 2B). In addition, there was a significant correlation between VEGF-C production and histamine secretion (r = 0.82; *p* < 0.001) (Figure 2C) and between VEGF-A and VEGF-C production (r = 0.64; *p* < 0.01) (Figure 2D).

### 3.3. Effects of IL-33 on the Release of Angiogenic and Lymphangiogenic Factors from HLMCs

**Figure 3:**
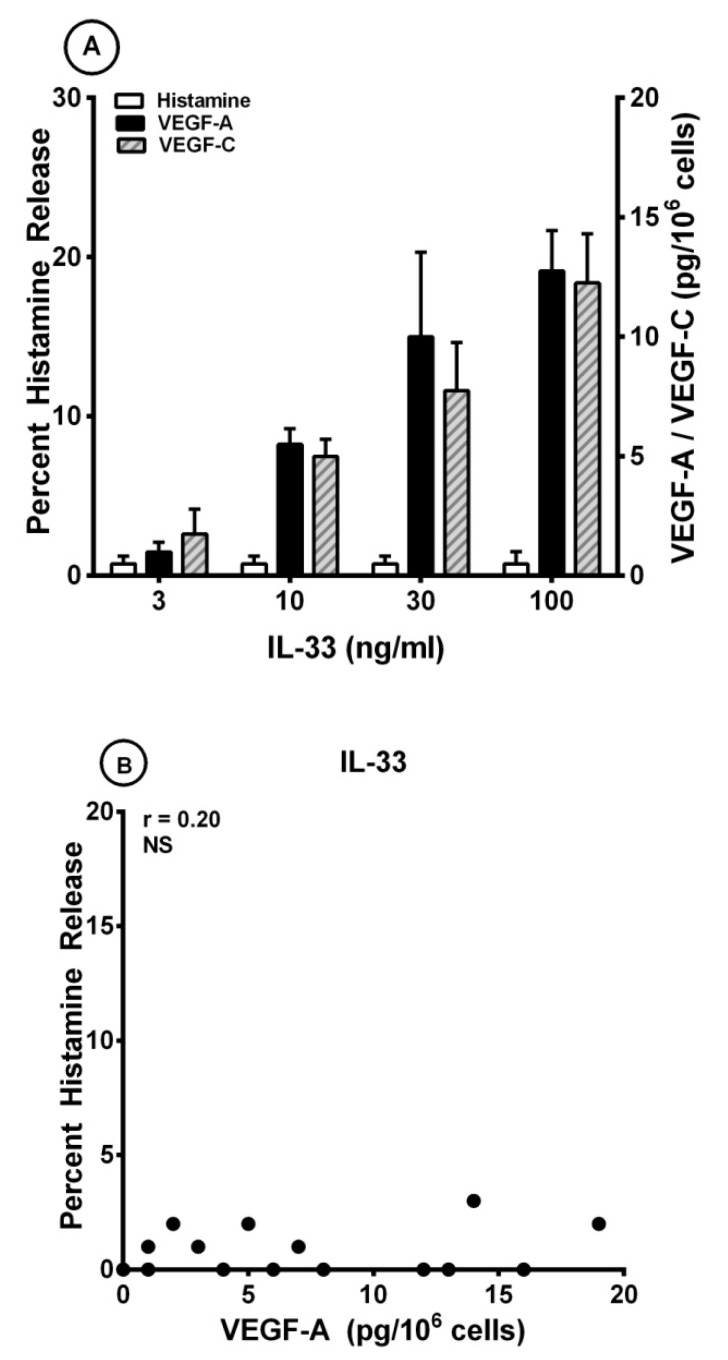

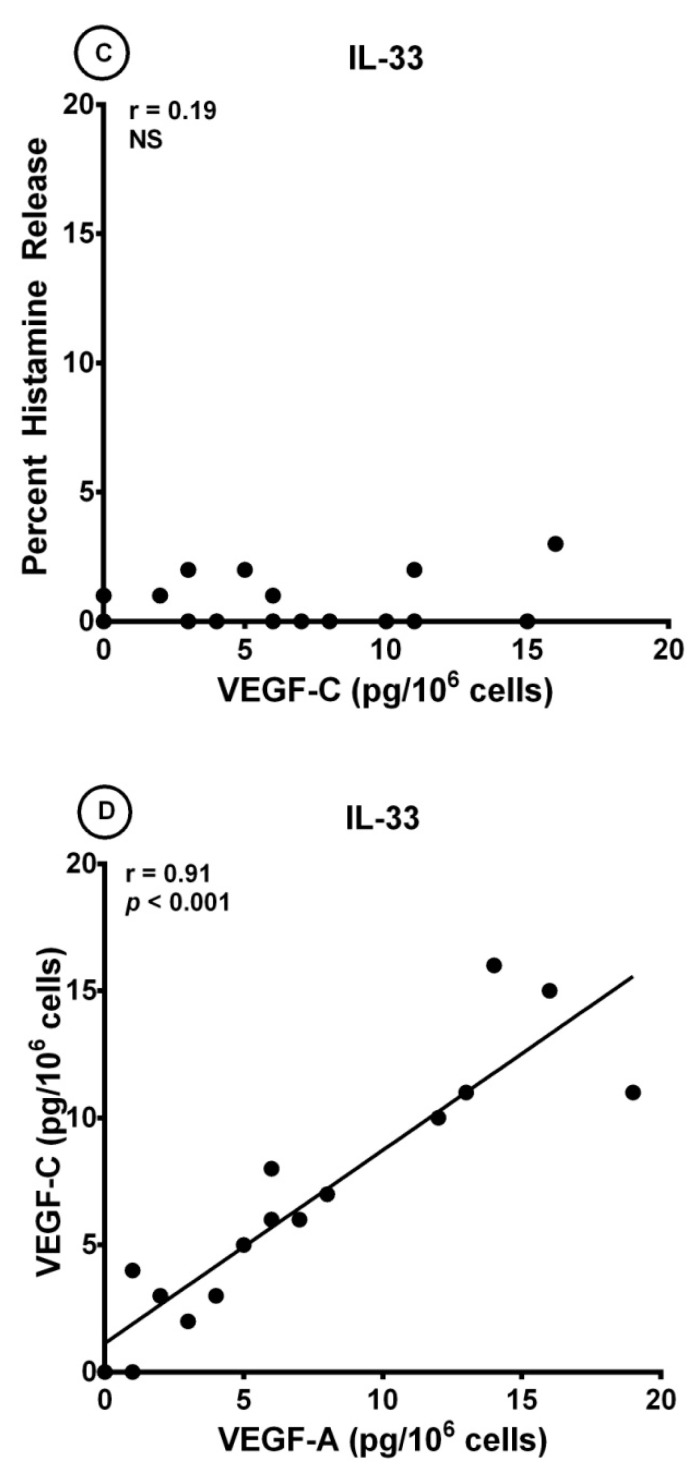
(**A**) Effects of increasing concentrations of IL-33 on histamine release and the production of VEGF-A and VEGF-C from four different preparations of human lung mast cells (HLMCs). HLMCs were incubated (45 min at 37 °C) with the indicated concentrations of IL-33 for histamine secretion or (12 h at 37 °C) for VEGF-A and VEGF-C release. Each bar is the mean ± SEM; (**B**) Lack of correlation (r = 0.20; NS) between VEGF-A release and the percent histamine secretion caused by IL-33 from HLMCs; (**C**) Lack of correlation (r = 0.19; NS) between VEGF-C release and the percent histamine secretion caused by IL-33 from HLMCs; (**D**) Correlation (r = 0.91; *p* < 0.001) between VEGF-A and VEGF-C release caused by IL-33 from HLMCs.

Several investigators have found that IL-33 can induce the release of different cytokines from human cord blood (CBMCs) and peripheral blood-derived mast cells (PBMCs) [17,68,69,70,71,78,99]. By contrast, IL-33 had no effect on the release of preformed mediators from mouse and human mast cells [100]. Interestingly, long-term incubation of human skin mast cells (HSMCs) with IL-33 reduced anti-IgE-induced histamine secretion from human skin mast cells (HSMCs) [68], whereas short-term exposure of HSMCs synergistically potentiated β-hexosaminidase release induced by substance P (SP) and anti-IgE [74]. Figure 3A shows that IL-33 (10 to 100 ng/mL) caused a concentration-dependent release of both VEGF-A and VEGF-C from HLMCs. By contrast, short-term incubation of HLMCs with IL-33 did not induce histamine release from these cells. The maximum VEGF-A release induced by IL-33 was significantly lower than that caused by both anti-IgE (12.7 ± 1.7 pg/10^6^ cells vs. 52.3 ± 3.3 pg/10^6^ cells; *p* < 0.001) and protein A (12.7 ± 1.7 pg/10^6^ cells vs. 24.3 ± 1.9 pg/10^6^ cells; *p* < 0.01). Similarly, the maximum release of VEGF-C induced by IL-33 was lower than that caused by anti-IgE (12.3 ± 2.1 pg/10^6^ cells vs. 49.0 ± 5.9 pg/10^6^ cells; *p* < 0.01) and protein A (12.3 ± 2.1 pg/10^6^ cells vs. 26.8 ± 2.6 pg/10^6^ cells; *p* < 0.01). There was no correlation between the production of both VEGF-A (Figure 3B) and VEGF-C (Figure 3C) and histamine release induced by IL-33 from HLMCs. By contrast, there was a significant correlation between the release of VEGF-A and VEGF-C (r = 0.91; *p* < 0.001) induced by IL-33 from HLMCs (Figure 3D).

### 3.4. Effect of Short-Term Priming by IL-33 on Superantigenic Release of Mediators from HLMCs

**Figure 4:**
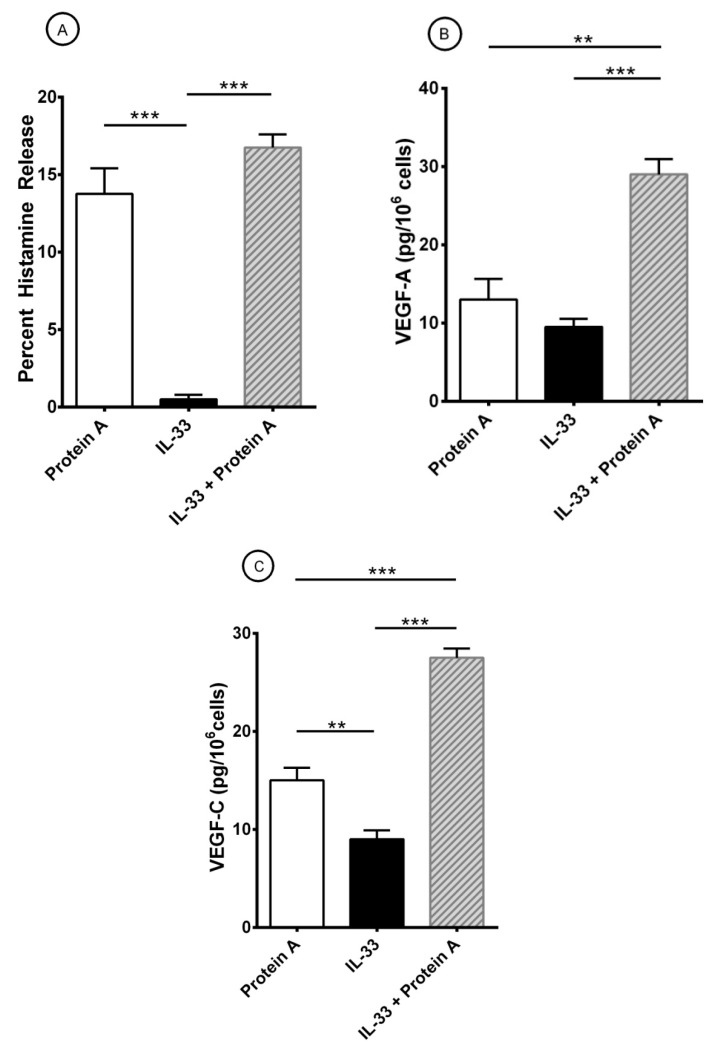
Effects of short-term priming by IL-33 an protein A-induced release of histamine (**A**), VEGF-A (**B**), and VEGF-C (**C**) from HLMCs. Mast cells were preincubated (30 min at 37 °C) with IL-33 (30 ng/mL) before exposure to protein A (100 nM). After 45 min incubation at 37 °C, HLMCs were harvested, centrifuged, and histamine release measured in the supernatants. For the evaluation of VEGFs, HLMCs were incubated for 12 h at 37 °C. At the end of incubation, cells were harvested, centrifuged and VEGF-A and VEGF-C measured in the supernatants. Results show the mean ± SEM obtained in three experiments. **p < 0.01; *** *p* < 0.001.

It has been reported that short-term priming by IL-33 can potentiate the release of cytokines induced by different stimuli from mouse [73] and human mast cells [17,20,69,71,74,101]. In a series of experiments, HLMCs were preincubated (30 min, 37 °C) with IL-33 (30 ng/mL) before exposure to protein A (100 nM). The results presented in Figure 4A confirm that IL-33 alone has no effect on histamine release whereas it induced both VEGF-A (Figure 4B) and VEGF-C (Figure 4C) from HLMCs. Interestingly, short-term priming by IL-33 potentiated the release of VEGF-A (Figure 4B) and VEGF-C (Figure 4C) induced by protein A from HLMCs.

**Figure 5:**
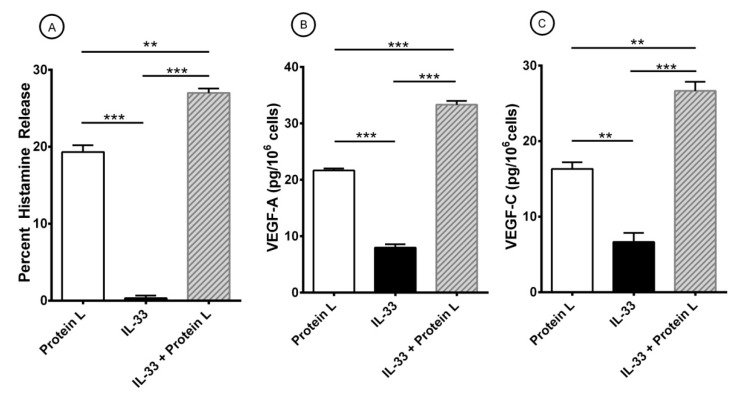
Effects of short-term priming by IL-33 and protein L-induced release of histamine (**A**), VEGF-A (**B**), and VEGF-C (**C**) from HLMCs. Mast cells were preincubated (30 min at 37 °C) with IL-33 (30 ng/ml) before exposure to protein L (100 nM). After 45 min incubation at 37 °C, HLMCs were harvested, centrifuged, and histamine release measured in the supernatants. For the evaluation of VEGFs, HLMCs were incubated for 12 h at 37 °C. At the end of incubation, cells were harvested, centrifuged and VEGF-A and VEGF-C measured in the supernatants. Results show the mean ± SEM of triplicate determinations obtained in a typical experiment. ** *p* < 0.01; *** *p* < 0.001.

Protein L is another immunoglobulin SAg, which binds to human Igs through a mechanism different from protein A [45,102]. This protein binds with high affinity (~10^10^ M^−1^) only to subtypes (V_K_I, V_K_III, and V_K_IV) of κ light chains of human Igs, including IgE [46,48,49]. We have found that protein L is a superantigenic stimulus inducing the release of preformed and the de novo synthesized inflammatory mediators from human cardiac mast cells through the interaction with κ light chains of IgE [98]. In a series of preliminary experiments, we found that increasing concentrations (1–300 nM) of protein L caused the release of inflammatory mediators from HLMCs (data not shown). Table 4 shows that protein L (100 nM) induced histamine release from HLMCs. Preincubation of protein L with increasing concentrations (0-3-1 μg/mL) of human monoclonal IgE λ light chain did not modify the activating property of protein L. By contrast, preincubation with the same concentrations of human monoclonal IgE κ light chain concentration-dependently inhibits the release of histamine induced by protein L from HLMCs. These results are compatible with the hypothesis that protein L activates HLMCs through the interaction with the κ light chain of IgE on lung mast cells. We then examined the interactions between protein L and IL-33 on the activation of HLMCs. Figure 5 shows the results of a typical experiment showing that protein L induces the release of histamine from HLMCs (Figure 5A). By contrast, IL-33 did not induce histamine secretion from HLMCs (Figure 5A) but caused a small production of VEGF-A (Figure 5B) and VEGF-C (Figure 5C). Short-term incubation (30 min, 37 °C) of HLMCs with IL-33 (30 mg/mL) potentiated the secretion of histamine (Figure 5A) and the release of VEGF-A (Figure 5B) and VEGF-C (Figure 5C) induced by protein L from HLMCs. Similar results were obtained in two additional experiments with different preparations of HLMCs (data not shown).

## 4. Discussion

Primary mast cells isolated from human lung parenchyma can be activated by a human IgG anti-IgE isolated from a patient with atopic dermatitis to release histamine, VEGF-A, and VEGF-C. Similar results were obtained by activating HLMCs with a monoclonal antibody anti-FcεRI. These findings indicate that HLMCs expressing FcεRI bind IgE, which is a central immunoglobulin in the pathogenesis of several allergic disorders [103,104] and pulmonary diseases [6,13]. Two bacterial superantigens, protein A and protein L, which bind to distinct regions of human IgE [43,44,45,49], activate HLMCs to release inflammatory (histamine), angiogenic (VEGF-A) and lymphangiogenic (VEGF-C) factors. IL-33, expressed and released by lung epithelial cells and endothelial cells [63,77], does not induce histamine release from HLMCs, but potentiates the release of VEGF-A and VEGF-C induced by superantigens from HLMCs.

Mast cells are strategically located in different compartments of human lung [3,4,78]. These cells and their mediators play a central role in the pathophysiology of bronchial asthma [1,2,9], lung remodeling [105], angiogenesis [14,16,17,98], lymphangiogenesis [14,18,94,98], chronic obstructive pulmonary disease (COPD) [106], and lung cancer [107,108].

Asthma is a heterogeneous syndrome that has been subdivided into phenotypes and molecular endotypes [109]. Type 2 (T2)-high subtype asthma is characterized by IgE-mediated activation of lung mast cells and includes the majority of asthmatic patients [103,104]. T2-low asthma is less well-characterized and presumably includes different clinical and genetic variants [110,111]. T2-low asthma may be driven by abnormal neuronal activation, structural abnormalities involving airway smooth muscle as well as bacterial and viral superantigens [112,113]. We found that cross-linking the IgE-FcεRI network on HLMCs induces the release of histamine. More importantly, we found that IgE-mediated activation of primary HLMCs also induces the production of VEGF-A and VEGF-C. The latter findings extend previous results indicating that activation of different types of human mast cells causes the release of VEGF-A [14,16,17,94,98]. Interestingly, IgE-mediated activation of HLMCs induces the release of VEGF-C, which is the most potent lymphangiogenic factor [114,115].

Several investigators have provided evidence that bacterial superantigens play a role in different allergic disorders [40,116]. We found that low concentrations of Staphylococcal protein A induces the release of histamine, VEGF-A, and VEGF-C from HLMCs. These findings might be relevant to explain the role of bacterial superantigens in the pathogenesis of various allergic disorders. The role of *S. aureus* in the pathogenesis of allergic diseases has been attributed to its capacity to activate T and B cells, resulting in cell proliferation and massive cytokine release [117]. On the other hand, it has been shown that Staphylococcal superantigens can induce the formation of IgE antibodies [95,118] and the presence of specific IgE has been associated with the severity of airway and skin allergic disorders [52,119,120]. There is also evidence that *S. aureus* can trigger the production of cytokines from human mast cells through the engagement of TLR2 and CD48 molecules [97]. Our results indicate that protein A might contribute to the role played by *S. aureus* in allergic diseases by inducing the release of histamine and angiogenic and lymphangiogenic factors from HLMCs through the interaction with the V_H_3 region of IgE.

Protein L is another Ig superantigen which specifically interacts with high affinity with the κ light chains of human Igs, including IgE [42]. In this study, we found that protein L is a potent stimulus to induce the secretion of histamine and the release of VEGF-A and VEGF-C from HLMCs. These results extend previous findings indicating that protein L induced the release of preformed (i.e., histamine) and de novo synthesized mediators (i.e., prostaglandin D_2_: PGD_2_) from human cardiac mast cells [98]. Collectively, these findings indicate that protein L is a complete mast cell secretagogue capable of releasing inflammatory, angiogenic and lymphangiogenic mediators implicated in cardio-pulmonary pathophysiology [121,122,123].

Recent studies revealed that cytokines such as thymic stromal lymphopoietin (TSLP) [124,125,126], IL-33 [78,99,127,128,129], and IL-25 [130], highly expressed in the airway epithelium, are implicated in human asthma [131]. These upstream cytokines serve as key regulators of T2-high and T2-low asthma [99,128,132]. In particular, IL-33 is overexpressed by epithelial cells in bronchial asthma [129] and activates different types of rodent [70,73,133] and human mast cells [17,20,68,69,71,72,74]. There is some evidence that IL-33, but not TSLP or IL-25, is central in models of allergic sensitization [78,127]. In this study, short-term incubation of HLMCs with IL-33 does not induce the secretion of histamine, confirming the results of previous studies [99]. By contrast, it has been reported that long-term (24 h) incubation of HLMCs with IL-33 caused marginal, but significant histamine release [99].

IL-33 can induce the production of several cytokines from mouse [70,73,133], human CBMCs [17,78] and PBMCs [20,69,72], mast cell lines [17,71], and primary mast cells [99]. We extended these findings by showing that IL-33 alone induces the release of angiogenic VEGF-A and lymphangiogenic VEGF-C from HLMCs. The activating property of IL-33 is likely mediated by the engagement of IL-33 receptor (ST2), which is highly expressed by human mast cells [72,99].

Angiogenesis plays a role in pulmonary pathophysiology [18,134,135]. VEGF-A is a major mediator of angiogenesis and can be produced by several immune cells [14,36,37,136,137]. To our knowledge, this is the first evidence that superantigens protein A and protein L induce the release of angiogenic factors from HLMCs raising the possibility that these cells can contribute to angiogenesis, a process of pivotal relevance in bronchial asthma [18,134] and lung cancer [135]. Further studies are needed to comprehensively define the contributive role of IL-33 and superantigens to angiogenesis in pulmonary disorders.

The mammalian lung is rich in lymphatic vessels [138] which are increased in human lung following infections [139,140,141]. We provide the first evidence that a superantigenic activation of HLMCs leads to the production of VEGF-C, a major mediator of lymphangiogenesis [142]. Lymphangiogenesis is canonically considered pivotal for the diffusion of metastasis to draining lymph nodes [143,144]. However, recent evidences indicate that VEGF-C can potentially exert protective effects, since inflammation-associated lymphangiogenesis can improve the resolution of inflammation [115,145]. Therefore, the contribution of bacterial superantigens to lung mast cell-mediated lymphangiogenesis requires additional investigations.

To the best of our knowledge, we provide the first evidence that IL-33 can induce the release of the lymphangiogenic factor VEGF-C from HLMCs. This finding extends a previous observation indicating that immunologically-activated human cardiac mast cells release VEGF-C [98]. The production of VEGF-C by activated primary human mast cells is intriguing because these cells are at the interface of the lymphatic and immune systems [146]. In several clinical and experimental studies, mast cells play a pro-tumorigenic role, whereas in others, they play an anti-tumorigenic role [25,26,143,147]. VEGF-C is mostly viewed as the most potent lymphangiogenic factor [115] controlling the formation of metastasis. However, increasing evidences indicate that, under certain circumstances, lymphangiogenesis and VEGF-C have protective effects in cancer [148]. Moreover, VEGF-C can exert a protective role in several inflammatory disorders [149,150] by favoring the resolution of inflammation [142,151,152]. The pathophysiological role of VEGF-C released by human lung and cardiac mast cells [98] deserves further investigations.

Several studies have provided evidence that IL-33 can exert a priming effect on the activation of rodent [73] and human mast cells [17,20,69,71,74]. A previous study reported that preincubation of LAD2 cells and CBMCs with IL-33 augmented SP-induced VEGF-A mRNA and VEGF-A protein secretion [17]. In our study, we found that short-term incubation of HLMCs with IL-33 potentiates the release of histamine, VEGF-A, and VEGF-C induced by superantigens protein A and protein L. The interactions between IL-33 and IgE-mediated stimuli (i.e., protein A and protein L) is unlikely mediated by an overexpression of FcεRI because it has been demonstrated that IL-33 does not increase FcεRI expression on human mast cells [74].

IL-33 is an alarmin overexpressed in lung epithelial cells [99,129] in asthmatic patients. IL-33 expression has been also identified in human airway smooth muscle (ASM) and HLMCs in mild-to-moderate asthma [99]. Using quantitative morphometry of the airway wall, it has been demonstrated that IL-33 causes a shift in mast cells from the submucosa to the airway epithelium associated with type 2 inflammation and airway hyperresponsiveness (AHR) [78]. A recent study highlighted a novel pathogenetic mechanism of interaction between IL-33 and *S. aureus* as inducers of airway inflammation in mice [118]. Intratracheal exposure to *S. aureus* derived serine protease-like protein (Spl) D upregulated IL-33 production in the lung leading to eosinophilia, bronchial hyperreactivity, and goblet cell hyperplasia in the airways. Interestingly, blocking IL-33 activity with a soluble ST2 receptor significantly reduced airway inflammation. Our findings highlight a novel immunologic mechanism by which IL-33 and superantigen protein A can amplify the immune response in inflammatory disorders involving lung mast cells. Collectively, these findings might have translational relevance emphasizing the relevance of IL-33/*S. aureus*-derived proteins in inducing inflammatory response in the airways [78,99,153]. The translational significance of the interactions between IL-33 and bacterial superantigens deserves further investigations in experimental models of asthma.

Our study has several limitations that should be pointed out. The in vitro experiments were performed using primary mast cells purified from lung parenchyma of patients undergoing thoracic surgery for lung cancer. The purity of HLMCs used in these experiments ranged from 49% to 81%. Although the activating properties of protein A, protein L and IL-33 were not affected by mast cell purity, we cannot exclude the possibility that contaminating cells may have influenced some of our results. In addition, IL-33 is released by tumor cells [84] and by a myriad of immune and non-immune cells localized in human lung [64]. There is the possibility that in vivo exposure of HLMCs to IL-33 could explain some of our results. In addition, HLMCs, although obtained from macroscopically normal lung tissue, are in close proximity to lung cancer cells. There is the possibility that the in vivo exposure to altered tumor microenvironment, such as low pH [154], hypoxia [155,156], lactate [157], or adenosine [37,158,159], may have affected the phenotypic expression and the functional activity of pulmonary mast cells.

In conclusion, our results indicate that two immunoglobulin superantigens, protein A and protein L, can interact with different domains of human IgE bound to FcεRI to induce the release of inflammatory, angiogenic and lymphangiogenic factors from human lung mast cells. IL-33 synergistically potentiates the superantigenic release of mediators from these cells. Future studies are needed to investigate whether these in vitro observations can help to understand the in vivo interactions between IL-33 and *S. aureus* in inflammatory airway disorders.

## Figures and Tables

**Table 1:** Effects of increasing concentrations of monoclonal antibody anti-FcεRI on histamine release and the production of VEGF-A (angiogenic) and VEGF-C (lymphangiogenic) from human lung mast cells.

Monoclonal Antibody (mAb) Anti-FcεRI (μg/mL)				
	10^−1^	1		3
**Percent Histamine Release**	14.0 ± 3.21		23.33 ± 4.80	24.66 ± 3.84
**VEGF-A** **(pg/10^6^ cells)**	16.33 ± 3.28		28.66 ± 1.45	51.33 ± 3.84
**VEGF-C** **(pg/10^6^ cells)**	16.33 ± 4.91		29.33 ± 6.43	45.0 ± 8.14

Human lung mast cells purified from three different donors were incubated with the indicated concentrations of mAb anti-FcεRI to evaluate histamine secretion (45 min at 37 °C) or VEGF-A and VEGF-C release (12 h at 37 °C). Each value is the mean ± SEM.

**Table 2:** Effect of human polyclonal IgG on histamine release and the production of VEGF-A and VEGF-C from human lung mast cells.

Human Polyclonal IgG (μg/mL)				
	10^−2^	10^−1^	1	3
**Percent Histamine Release**	1.66 ± 1.20	1.66 ± 0.88	1.33 ± 0.88	1.83 ± 1.01
**VEGF-A** **(pg/10^6^ cells)**	0.33 ± 0.33	2.30 ± 0.33	1.66 ± 1.20	0.66 ± 0.66
**VEGF-C** **(pg/10^6^ cells)**	1.16 ± 0.60	0.50 ± 0.50	1.4 ± 0.94	0.83 ± 0.44

Increasing concentrations of human polyclonal IgG purified from the serum of three healthy donors as described elsewhere [89] were incubated with three different preparations of human lung mast cells (HLMCs). HLMCs were incubated (45 min at 37 °C) with the indicated concentrations of IgG to evaluate histamine secretion or VEGF-A and VEGF-C release (12 h at 37 °C). Each value is the mean ± SEM.

**Table 3:** Effects of preincubation of protein A with human monoclonal IgM V_H_3^+^ or IgM V_H_6^+^ on the activation of HLMCs.

Stimulus	Percent Histamine Release
Protein A	18.3 ± 0.9
IgM V_H_3^+^	0.3 ± 0.3
IgM V_H_3^+^ + Protein A	3.0 ± 0.6 ***
IgM V_H_6^+^	0.7 ± 0.6
IgM V_H_6^+^ + Protein A	18.7 ± 0.3

Protein A (300 nM) was preincubated (15 min at 37 °C) with IgM V_H_3^+^ (10 μg/mL) or IgM V_H_6^+^ (10 μg/mL) and incubation continued for another 45 min at 37 °C. Results show the mean ± SEM of percent histamine release obtained from three experiments with different preparations of HLMCs. *** *p* < 0.001 when compared to protein A alone.

**Table 4:** Effects of preincubation of protein L with human monoclonal IgE λ or IgE κ on the activation of HLMCs.

Stimulus	Percent Histamine Release
Protein L (100 nM)	19.0 ± 1.5
IgE λ (0.3 μg/mL) + Protein L	18.7 ± 1.8
IgE λ (1 μg/mL) + Protein L	18.3 ± 2.0
IgE κ (0.3 μg/mL) + Protein L	13.7 ± 0.7 *
IgE κ (1 μg/mL) + Protein L	3.7 ± 1.2 **

Protein L (100 nM) was preincubated (15 min at 37 °C) with increasing concentrations (0.3 or 1 μg/mL) of human monoclonal IgE λ light chain or human monoclonal IgE κ light chain, and incubation continued for another 45 min at 37 °C. Results show the mean ± SEM of triplicate determinations of percent histamine release. Similar results were obtained in another experiment. * *p* < 0.05 when compared to protein L alone. ** *p* < 0.01 when compared to protein L alone.

## Data Availability

The data presented in this study are available on request from the corresponding author.

## Abbreviations

AHR	Airway hyperresponsiveness
ASM	Airway smooth muscle
BSA	Bovine serum albumin
COPD	Chronic obstructive pulmonary disease
CBMC	Cord blood-derived mast cell
FcεRI	High-affinity receptor for IgE
FCS	Fetal calf serum
H	Heavy
H-aIgE	Human IgG anti-IgE
HLMC	Human lung mast cell
Ig	Immunoglobulin
IL-33	Interleukin-33
IL	Interleukin
L	Light
LTC_4_	Cysteinyl leukotriene C_4_
mAb	Monoclonal antibody
*P. magnus*	*Peptostreptococcus magnus*
PBMC	Peripheral blood-derived mast cell
PGD_2_	Prostaglandin D_2_
*S. aureus*	*Staphylococcus aureus*
SAg	Superantigen
SE	*Staphylococcus aureus* enterotoxins
SP	Substance P
TCR	T cell receptor
Treg	regulatory T cell
TSLP	Thymic stromal lymphopoietin
V	Variable
VEGF	Vascular endothelial growth factor
